# Setting larger session duration goals is associated with greater future physical activity

**DOI:** 10.1371/journal.pone.0208644

**Published:** 2018-12-10

**Authors:** Ernestine G. Jennings, Shira I. Dunsiger, Beth C. Bock, Sheri J. Hartman, David M. Williams, Bess H. Marcus

**Affiliations:** 1 Centers for Behavioral & Preventive Medicine, The Miriam Hospital, Providence, Rhode Island, United States of America; 2 Department of Psychiatry & Human Behavior, Alpert Medical School of Brown University, Providence, Rhode Island, United States of America; 3 Department of Behavioral & Social Sciences, Brown University School of Public Health, Providence, Rhode Island, United States of America; 4 Department of Family Medicine and Public Health, University of California San Diego, La Jolla, California, United States of America; 5 Moores Cancer Center, University of California San Diego, La Jolla, California, United States of America; Curtin University, AUSTRALIA

## Abstract

Many national (US) and International guidelines for physical activity provide guidance that under-active and sedentary adults can begin by accumulating moderate-to-vigorous physical activity (MVPA) in bouts as brief as 10 minutes. This guidance fits well with Goal Setting theory in that goals should be realistic and achievable, and is also consistent with Social Cognitive theory since achieving small goals should boost self-efficacy and thus, encourage continued physical activity. In contrast, Behavioral Economics might suggest that fewer, longer bouts would be more conducive to the adoption of physical activity due to the costs incurred with each separate bout of MVPA. This paper examines patterns of MVPA adoption among a sample of under-active adults from the perspective of goal setting theory and behavioral economics to explore specific strategies to help people who are in the early stages of PA activity adoption. Under-active men and women (N = 225; mean age = 46 ± 10; mean BMI = 28 ± 4.48) who enrolled in a PA intervention participated in a single goal setting session at enrollment. Participants were encouraged to set realistic goals and to increase their activity to meet national recommendations (150 minutes/week of moderate to vigorous physical activity [MVPA]) by the 6-month follow up. This process included identifying a specific frequency goal (days/week) and session duration goal (minutes/day). At baseline, participants reported average weekly MVPA of 14.59 min (± 24), which increased to an average of 140.52 (± 143.55) at 6 months. MVPA goals at baseline averaged 33.24 min/day (± 18.08) and 3.85 days/week (± 1.31). Analyses showed that longer session duration goals set at baseline were associated with more weekly minutes of MVPA at 6 months (b = 1.26, SE = 0.58, t = 2.17, p = 0.03). There was no significant association between goals for number of days per week (frequency) or total minutes of weekly MVPA (minutes x frequency) and MVPA at 6 months. Widely promoted guidelines for uptake of physical activity recommend accumulating physical activity in bouts as short as 10 minutes. This recommendation may ultimately hinder the adoption of physical activity among under-active and sedentary individuals. For the purposes of behavioral adoption of MVPA, more ambitious session duration goals appear to result in higher levels of physical activity participation.

## Introduction

Currently one third of American adults report no regular physical activity (PA) during their leisure time and less than half are meeting the current national guidelines for aerobic activity [1]. Low levels of moderate-to-vigorous physical activity (MVPA) among American adults constitute a major public health problem, in that participation in regular MVPA has significant health benefits including reduced risk of all-cause mortality [2, 3], cardiovascular disease [2, 3], diabetes [4], depression [5], osteoporosis [6], and cancers of the breast [7] and colon [8]. MVPA also enhances weight loss and has been shown to prevent weight regain [9–11].

The association between MVPA and health benefits has led to multiple reports and position papers from public health, medical, and scientific groups on the importance of promoting MVPA [9, 12–20]. The American College of Sports Medicine (ACSM), American Heart Association (AHA), World Health Organization (WHO), and the U.S. Centers for Disease Control and Prevention (CDC) have each recommended that most adults should engage in moderate-intensity physical activity for a minimum of 30 minutes on five days weekly or vigorous-intensity physical activity for a minimum of 20 minutes thrice weekly. Combinations of moderate- and vigorous-intensity activity can be performed to achieve similar energy expenditure (500–1000 MET minutes per week) [9, 17, 18]. Each of these guidelines includes a recommendation that MVPA can be accumulated in short bouts of as little as 10 minutes [9, 12–20]. More recent guidelines from the Physical Activity Guideline Advisory committee recommend even shorter bouts of activity and that additional health benefits are gained by doing physical activity up to 300 minutes of MVPA [21].

The recommendation that exercise may be acquired in bouts of as little as 10 minutes [9, 12, 14, 15, 17, 18] is detailed in the position paper from the AHA/ACSM [10] and is listed as a Class I recommendation (“c*onditions for which there is evidence and/or general agreement that a given procedure or treatment is useful and effective”)*. A footnote was included indicating these data were derived either from a single randomized trial or from multiple non-randomized studies. Similarly, the U.S. Surgeon General’s report [17] suggests that very sedentary individuals begin with daily bouts of MVPA as small as 5 minutes, performed across various times across the day. The report also notes that this approach is based primarily on expert opinion and clinical experience, since the benefits and risks of various approaches to MVPA initiation for sedentary/unfit persons have not been systematically evaluated [17]. However, regardless of the physiological benefits of short bouts of MVPA, there is little data on the efficacy of accumulating MVPA in short bouts as a behavioral strategy to promote MVPA adoption among sedentary individuals.

The notion of beginning with short bouts of MVPA is intuitively appealing and fits well with both Goal Setting and Social Cognitive theories. While specific strategies used to set behavioral goals vary, most approaches address key components, ensuring that goals are SMART: Specific, Measurable, Achievable, Realistic, and Time-bound [22]. Setting specific goals should provide a strategy for organizing MVPA information and skills into practical, manageable steps [23], and helps direct efforts toward goal-relevant activities [24]. However, a systematic review of the literature found that specific goals were no more effective at increasing MVPA than more vaguely defined goals (e.g., ‘to exercise more’) [25]. Similarly, although guidelines for MVPA often center on specific weekly targets [18], empirical studies indicate that weekly goals are less effective than either daily or daily plus weekly goals [25]. Setting goals for short bouts of MVPA (e.g., as little as 10 minutes) also fits well within Social Cognitive Theory [26, 27] in that setting small, achievable goals should increase self-efficacy and thereby encourage continued participation in MVPA.

In contrast, Behavioral Economics [28, 29] may suggest that breaking MVPA into smaller, more frequent bouts would hinder physical activity adoption rather than promote it. Behavioral Economics posits that contextual factors including the relative costs of engaging in any activity must be taken into account to understand how and why an individual engages (or does not engage) in any activity. In the context of MVPA, costs to be considered include those associated with preparing to be active (e.g., is special clothing or equipment needed), time constraints (e.g., competing demands), opportunity costs (alternative ways of spending time), and costs associated with returning to other activities (e.g., showering, travel time to work/home). To the degree that any or all of these costs are associated with the chosen type of physical activity, the costs rise with the number of bouts regardless of their duration. Therefore, one would anticipate that longer bouts of MVPA would result in greater physical activity compared to frequent, shorter bouts.

The goal of this paper is to examine which part(s) of an exercise goal (session duration, frequency, and/or total time) are associated with increased participation in MVPA among people who are in the early stages of physical activity adoption.

## Methods

### Study design and participants

All participants provided written informed consent approved by the Institutional Review Board of the Miriam Hospital (IRB registration #TMH IRB-00000482) and this research was conducted according to the principles expressed in the Declaration of Helsinki. Data for this study were collected from September 2006—November 2009 with analysis for this paper being completed in May 2014. Details of the parent study design and major outcomes have been published elsewhere [30, 31]. The parent trial is registered at ClinicalTrials.gov (#NCT 00367029).

We conducted a secondary data analysis from a study of 225 healthy, under active, (<90 min/week of MVPA at program entry) adults randomized to one of two tailored print-based physical activity interventions [31]. Participants were recruited through newspaper advertisements, online postings, and fliers. At baseline, participants were randomized to receive either: (a) a previously validated print-based program [32], with individual tailoring based on five psychosocial constructs from the Transtheoretical model and Social Cognitive Theory (*Print*); or (b) an enhanced intervention, with tailoring based on five additional constructs from Social Cognitive Theory (*Enhanced Print*). At baseline, prior to randomization, all participants received a 15-minute, face-to-face counseling session to set specific physical activity goals for themselves. While participants were informed that the overall aim of the intervention was to help them increase their MVPA to a level that met or exceeded national (US) recommendations (150 min/week of MVPA) [17] by six months, they were also instructed that they could select goals that were more or less than those recommendations. The goal-setting process included setting a specific goal for number of minutes per day (session duration) and days per week (frequency) throughout the first six months of the study. Participants were encouraged to set realistic, challenging and specific goals, to write down their goal and to make specific plans for how they would meet their goal (e.g., take a walk every day at lunch hour) [33].

### Measures

#### Physical activity goals

We obtained the participant’s anticipated MVPA session duration goals and frequency goals from the baseline goal-setting session interview. We then calculated the total anticipated MVPA time expressed as minutes/week of anticipated MVPA from the session duration and frequency goals set by each participant (duration x frequency).

#### Physical activity behavior

Participation in MVPA was assessed at baseline, six months and 12 months, with six months as the primary outcome for the study. Data were collected by trained research staff using the interviewer-administered 7-day physical activity recall questionnaire (PAR). The PAR is a previously validated questionnaire administered by trained interviewers and includes querying individuals on their cumulative sleep and moderate, hard, and very hard physical activities over the previous seven days [34]. Consistent with previous research [34] time spent in moderate, hard, and very hard activities were only included if reported in continuous bouts of at least 10 minutes. To improve the accuracy of self-report, all participants underwent a 10-minute treadmill exercise to demonstrate moderate-intensity physical activity, based on the participant’s age predicted maximal heart rate, prior to the PAR interview [35].

### Analyses

Analysis included all participants who attended the baseline goal setting session (N = 225). Descriptive statistics including baseline demographics and goal setting behavior are presented in Table 1. For this paper interest is on the aggregated study sample, therefore between-group differences are not presented [31]. For the primary outcome, (min/week of MVPA) unadjusted means and standard deviations were summarized at baseline and six month follow-up.

**Table 1:** Baseline characteristics of study sample (N = 225).

	Overall Sample (N = 225)
Body Mass Index	28.01 (4.48)
Age (years)	46.88 (10.01)
Gender (% Female)	88.44% (N = 199)
Education (% at least some college)	61.16% (N = 137)
Employment (% employed)	83.04% (N = 186)
Marital Status (% Married/Partnered)	63.84% (N = 143)
Days/Week of Anticipated Activity	3.85 (1.31)
Mins/Day of Anticipated Activity	33.24 (18.08)
Baseline Physical Activity (min/week)	14.59 (24.00)

Means and standard deviations (SD) are presented unless noted otherwise. Days/week of anticipated activity and mins/day of anticipated activity were collected as part of the goal setting session. Baseline Physical activity was the subjectively reported baseline value of the outcome variable (as measured by the 7-day PAR).

Using a generalized linear model (GLM), we examined the association between baseline goals and minutes/week of MVPA at the six month follow-up, controlling for baseline values, and covariates chosen *a priori* (treatment assigned, gender, employment status and season) that represent potential confounders. Goals were coded using two variables to capture separate effects of frequency and duration. Analyses were conducted on the intent-to-treat sample, including all participants randomized at baseline. GLMs use a likelihood-based approach to estimation and therefore made use of all available data without directly imputing missing values. Residual diagnostics and influence statistics were used to assess model fit.

Next, we examined whether goals set at baseline were associated with the probability of meeting the participant’s goal for total weekly minutes of MVPA (Anticipated Total Physical Activity: ATPA) at six months (binary outcome) using a regression model implemented with Generalized Estimating Equations with robust standard errors [36]. Specifically, we regressed binary indicators of meeting the calculated MVPA goal (frequency x duration) on specific goals (frequency and duration) set at baseline, treatment, baseline level of MVPA, gender, employment status and season, using binomial errors, a *logit* link function and a working unstructured correlation to accommodate within-subject correlation. Since the intervention aim was to increase weekly time spent in MVPA, we did not break down ATPA into two separate predictors (frequency and duration), but rather, focused on the calculated total minutes over the week. Meeting the ATPA goal was defined as reporting at least 80% of the total minutes/week of MVPA at six months that was set as a goal at baseline. Non-linear effects of goals set were also examined. In the case of any missing six month outcomes, we tested the effects of goals set under the assumption that missing = not meeting six month goals as well as among the sample of completers only.

Finally, using a generalized linear model, we examined the association between meeting the ATPA goal at six months and min/week of MVPA at 12 months, controlling for covariates. Residual diagnostics and influence statistics were used to assess model fit. All analyses were conducted in SAS 9.3 and significance level set *a priori* at 0.05.

## Results

The sample was predominantly female (88.4%) with average age of 46.9 years (SD = 10.01) and a mean body mass index (BMI) of 28.0 (SD = 4.48) at baseline. A full description of the aggregated sample of participants (N = 225) is provided in Table 1. Participants’ ATPA goals at baseline averaged 33.2 min/day (SD = 18.08, range 5–120), and 3.85 days/week (SD = 1.31, range 1–7) of MVPA by six months. At baseline, mean MVPA level was 14.59 min/week (SD = 24.00). MVPA increased to 140.52 min/week (SD = 143.55) at six months and 135.92 min/week (SD = 183.22) at 12 months.

Total minutes/week of anticipated MVPA (ATPA = frequency x duration) was not a significant predictor of MVPA at six months. Results indicate a significant positive association between session duration goals and minutes of MVPA reported at six months, such that mean MVPA at six months was significantly higher for those who set higher session duration goals at baseline (b = 1.26, SE = 0.58, t = 2.17, p = 0.03), with each increase in planned goal of 10 min/day corresponding to a 12 min/week increase in actual MVPA at six months. There was no significant association between frequency baseline goals (days per week) and six month activity levels (Table 2).

**Table 2:** Regression estimates from GLM of association between goals set at baseline and six month activity levels (N = 225).

	Parameter Estimate	Standard Error	t Value	P-value
Days/Week of Anticipated MVPA	0.35	8.21	0.04	0.97
Minutes/Day of Anticipated MVPA	1.26	0.58	2.17	0.03

Model controlled for baseline MVPA, treatment assigned, gender, employment status and season.

**Fig 1:**
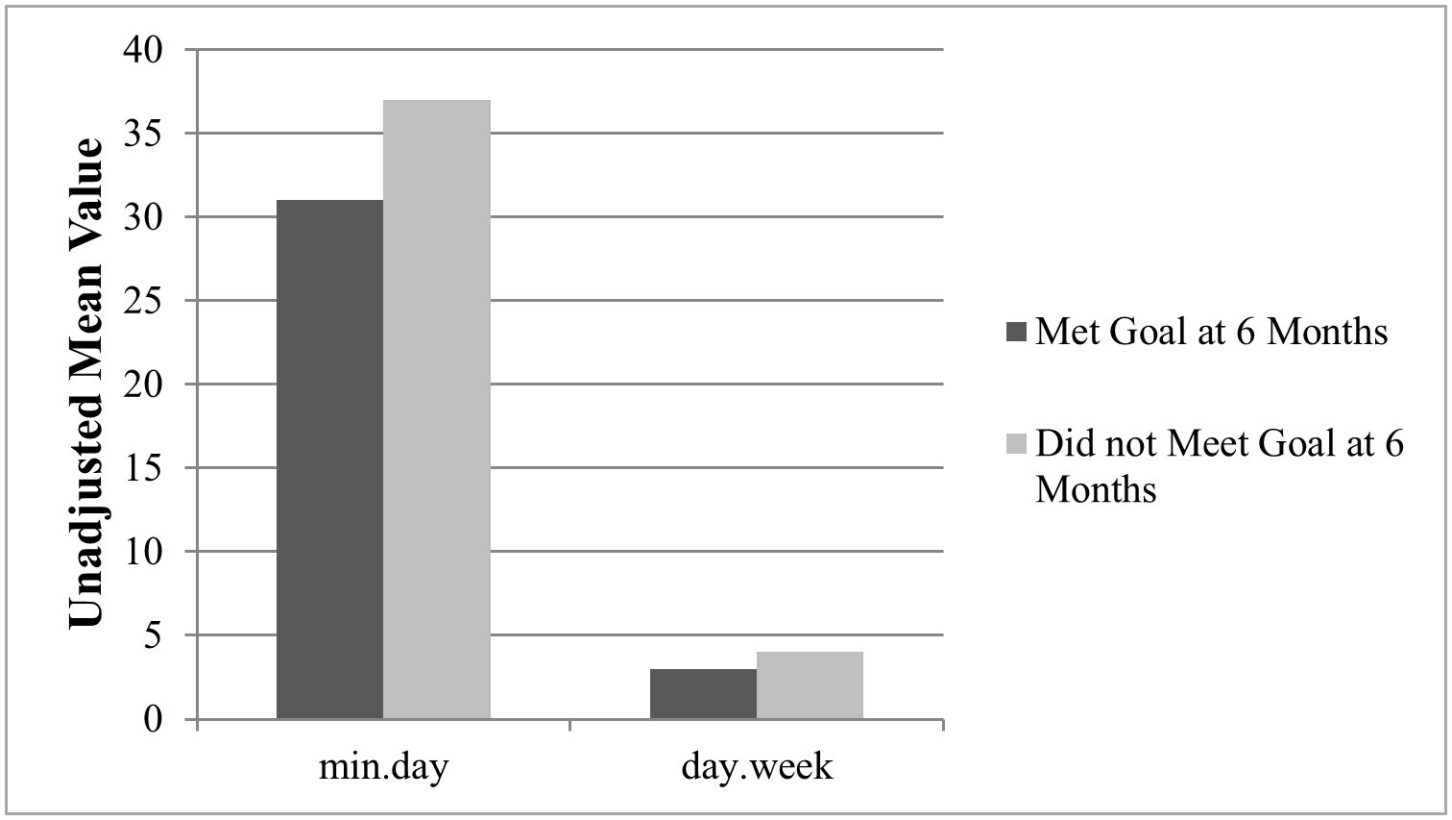
Physical activity participation at six months stratified by whether or not goals set at baseline were met.

Of the 198 participants (88% of baseline sample) who completed physical activity assessments at six months, 61.6% had met their initial goal for MVPA. Unadjusted mean goals at baseline are summarized for those who did and did not meet goals at six months in Fig 1. When controlling for covariates, there was a significant, negative association between initial goal and the odds of meeting that goal at the six months (b = -0.01, SE = 0.002, Z = -3.80, p < .01). Results suggest that each 30 min/week increase in total anticipated MVPA at baseline was associated with a 22% decrease in the odds of meeting that goal at six months (OR = 0.78, 95% CI: 0.69–0.89).

Finally, adjusted models indicate there was a significant association between meeting goals at six months and MVPA at 12 months (t = 3.17, p < .01), such that those who met their six month goal engaged in an average of 83.8 min/week more MVPA at 12 months compared to those not meeting their goal at six months. (SE = 26.45).

## Discussion

Results of this study indicate that adults who set larger session duration goals in terms of minutes of MVPA per day, engaged in more total weekly MVPA at follow up than those who set smaller session duration goals. Frequency goals (number of days per week), were not associated with MVPA outcomes. That is, whether the individual set a goal of engaging in MVPA on three days per week or seven had no impact on the total amount of MVPA performed.

Consistent with goal-setting theory [23, 33], individuals in this study who set smaller, more easily achieved goals were more likely to achieve those goals than those who set larger goals. However, results also suggest that for the overall aim of increasing total MVPA, it may be more effective to focus on setting larger *session duration* goals. That is, it may be better to fail to achieve a goal of 45 minutes/day by reaching only 30 minutes/day (for example), than to succeed in achieving a goal of 15 minutes/day.

Results also support the notion from Social Cognitive Theory that setting achievable goals builds self-efficacy and sets the stage for further success experiences. In this study, there was a significant association between meeting goals at six months and total minutes of MVPA at 12 months. Individuals who had smaller ATPA goals (frequency x duration) at baseline were more likely to achieve them at six months, and those who achieved their goals at six months went on to do more MVPA at 12 months than those who failed to achieve their 6-month goals. This creates a bit of a conundrum for interventionists. If we guide participants toward easily achievable goals it may increase self-efficacy, but produce less MVPA in the short run. However, guiding individuals toward more ambitious goals may increase MVPA more rapidly, but may also lead to less MVPA over longer periods of time if those goals are not achieved and self-efficacy suffers.

Recommendations for physical activity may be conceptualized in three domains: (1) physical activity needed for cardiovascular fitness, (2) physical activity to reduce health risks, and (3) physical activity strategies to promote behavior change. These domains do overlap, for example the amount of physical activity that is sufficient to reduce health risks may also improve cardiovascular fitness. However, there are also areas where these domains may be distinctly different. The amount and/or intensity of physical activity needed to improve the already high fitness performance of a marathon athlete, for example, may have no additional impact on his/her health risks. Likewise, the minimum necessary physical activity needed to make changes in cardiovascular performance (e.g., 10-minute bouts), may not be sufficient to support long-term behavioral adoption of MVPA among previously sedentary or under active individuals. Recommendations for physical activity adoption in the U.S. Surgeon General’s report [17], included protocols starting with bouts as small as 5–10 minutes. These recommendations make sense from the clinical perspective of trying to limit the risks of introducing physical activity among sedentary individuals, particularly those with comorbid health conditions. However, these recommendations were also based on expert opinion and/or clinical observation focusing on risks of engaging in physical activity, rather than on empirical studies of strategies for promoting physical activity behavior change. There is very little guidance for health professionals on the specific strategies on setting goals for the adoption and maintenance of regular physical activity.

Behavioral Economics [28, 29] may provide an alternate perspective for understanding the results obtained in this study. To the degree that costs are associated with each bout of physical activity (e.g., disengaging in a current activity, traveling to a gym or other facility, showering before returning to work, changing into different clothing), costs rise in direct proportion with the number of bouts of physical activity but are less influenced by the duration of any one bout. Thus, the context in which goals are set is important. Our participants’ success with setting larger session duration goals may be explained by this efficiency principle, in that people will do the most they can for the least cost. Unfortunately, we did not collect detailed data on the contextual costs of the participants’ physical activity choices and thus cannot draw any conclusions in this regard.

### Implications for practice

Results of this study may have implications for interventions designed to increase physical activity and the advice provided by health fitness professionals and providers to patients who need to become physically active. Results obtained in this study suggest that setting larger session duration goals is associated with greater minutes/week of MVPA at follow-up. Ultimately helping patients meet their fitness goals by helping them refine those goals and design a structured plan to help them achieve them. Thus, clinicians may consider encouraging patients to set larger session duration goals when starting a new physical activity routine. Additional goal-setting sessions may be needed during the first months of treatment to reinforce gains and ensure goals are achievable. However, as these results pertain to a single study, more research is needed that is designed specifically to examine the effects of different qualities of physical activity goals (e.g., duration, frequency, intensity, etc.) and across different populations (i.e., diverse age, gender and health status, etc.) prior to making definitive recommendations for clinical practice.

### Implications for research

Additional research is needed to answer several questions raised in this study. While results showed that goals set with respect to duration of activity (not frequency) are associated with successful adoption of MVPA, more research is needed to understand whether people are more likely to adopt a regular exercise routine if the focus is on the duration of activity versus the frequency of activity, and to understand both short-term and longer-term outcomes. Few studies report on individual bouts of MVPA, but rather, tend to report only daily or weekly totals—this is a major barrier to understanding the efficacy of short bouts of physical activity. The process goal setting with sedentary individuals needs additional research, particularly regarding the tension between small, easily achievable goals, and goals that are sufficiently challenging to produce meaningful, sustainable behavior change. While individuals may be successful at setting small goals, they do not ultimately meet the overall goal of increasing their activity to a level that would result in health benefits. Finally, additional work is needed to understand the relationship between the frequency of physical activity and health benefits (e.g., do the same benefits accrue for 30 minutes 5 days/week as for 75 minutes twice weekly?). Such research is needed to develop recommendations for setting goals that address both adoption and maintenance of physical activity and to improve overall health.

### Limitations

There are several limitations to consider in the context of these findings. Participants were not randomly assigned to larger versus smaller goals (frequency or session duration), thus we cannot make causal inferences regarding the association between achievement of MVPA and the size of goals set at baseline. Since participants were free to set large or small goals, the size of the calculated goal (total anticipated min/week), could have been a proxy measure of motivation. That is, individuals who were more motivated may have set higher goals, although it is not clear why more motivated individuals would have set higher session-duration goals and not larger frequency goals. This study did not measure motivation, thus we cannot assess the degree to which motivation may have been responsible for differences in goal setting. Similarly, all individuals in this study had responded to advertisements to join a physical activity study. Such persons may be different from other individuals, and results of this study should not be used to draw conclusions about the larger population of sedentary and under-active individuals. Potentially conducting a qualitative study would allow us to understand why these factors would impact goal setting.

### Conclusions

Among under-active individuals attempting to increase physical activity participation, setting larger session duration goals was associated with greater MVPA. The goals set regarding the number of days per week did not impact MVPA outcomes.

## Supporting information

S1 Dataset Original data set used in these analyses. (SAV)
